# Assessment of Pseudocoarctation of the Aorta with Saccular Aneurysms by Four-Dimensional Flow Magnetic Resonance Imaging and Histological Analysis

**DOI:** 10.3400/avd.cr.22-00077

**Published:** 2022-12-25

**Authors:** Hiromasa Ito, Yoshito Ogihara, Masaki Ishida, Hisato Ito, Kyoko Imanaka-Yoshida, Kaoru Dohi

**Affiliations:** 1 Department of Cardiology and Nephrology, Mie University Graduate School of Medicine, Tsu, Mie, Japan; 2 Department of Radiology, Mie University Graduate School of Medicine, Tsu, Mie, Japan; 3 Department of Thoracic and Cardiovascular Surgery, Mie University Graduate School of Medicine, Tsu, Mie, Japan; 4 Department of Pathology and Matrix Biology, Mie University Graduate School of Medicine, Tsu, Mie, Japan

**Keywords:** magnetic resonance imaging, thoracic aortic aneurysms, pathology

## Abstract

In this study, we present the case of a 21-year-old woman with pseudocoarctation of the aorta with saccular aneurysms that were evaluated by four-dimensional flow magnetic resonance imaging and histological analysis. We observed complete occupation of the aneurysm sacs by vortex flow and high peak wall shear stress in the proximal region of the kinked aorta. The aortic replacement was performed for the thoracic aortic aneurysms and the clinical course was uneventful. The aneurysms were histopathologically diagnosed as pseudoaneurysms based on the disappearance of all three layers and their replacement with collagen-rich connective tissues. These findings indicate that abnormal flow dynamics and the resulting abnormal shear stress in the aorta may play central roles in the formation and development of a saccular aneurysm.

## Introduction

Pseudocoarctation of the aorta (PCOA) is an infrequent congenital disorder that is characterized by kinking at the level of the ductus arteriosus without collateral circulation and pressure difference between the upper and lower limbs.^1)^ A lack of luminal narrowing is one of the significant features of PCOA and is usually asymptomatic that is accidentally found in an abnormal posterolateral chest radiograph. However, some cases develop aneurysmal formation, leading to sudden aortic rupture or dissection.

Several recent studies on the assessment of aortic diseases by four-dimensional flow magnetic resonance imaging (4D flow MRI) provide information about the association between hemodynamics values and aortic abnormalities.^2,3)^ Nonetheless, the use of 4D flow MRI analysis for detecting aneurysms associated with PCOA has not been previously reported. Herein, we report a case of a young female with a PCOA-related saccular aneurysm who underwent a 4D flow MRI and detailed pathological analysis.

## Case Report

**Figure 1:**
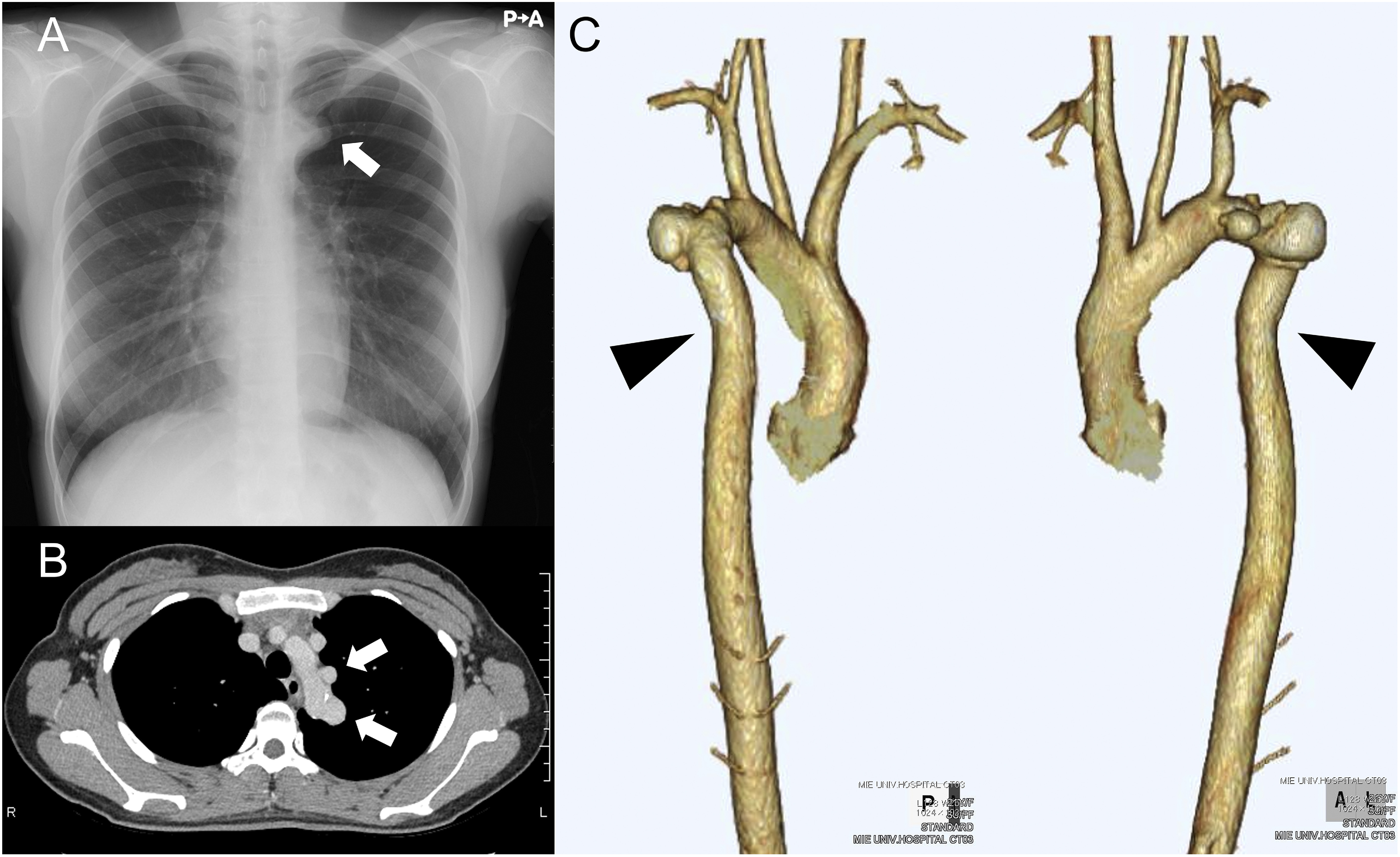
Fig. 1 (**A**) Chest X-ray image. The white arrow indicates the aneurysmal shadow of the aorta. (**B**) Cross-sectional computed tomography (CT) image of the saccular aneurysms (white arrows). (**C**) Three-dimensional CT angiographic images demonstrating the saccular aneurysms and kinking of the descending aorta (black arrowheads).

A 21-year-old woman free of trauma with an accidentally discovered abnormal shadow in the left superior mediastinum in her chest X-ray was referred to us (**Fig. 1A**). The patient had no history of medical illness or major traumatic injuries. Computed tomography (CT) angiography showed multiple saccular thoracic aortic aneurysms (TAAs) distal to the origin of the left subclavian artery (aortic isthmus) and mild kinking of the descending thoracic aorta (**Figs. 1B** and **1C**). The neck width and the sac depth of the majority of the aneurysms were 14 mm and 12.5 mm, respectively. In addition, the sac depth/neck ratio was 0.89. The right/left ankle-brachial pressure index was 1.00/1.00. Moreover, no blood pressure gradient between the upper and lower extremities was recorded. Hence, the patient was diagnosed with PCOA. The right/left brachial-ankle pulse wave velocity was 986/944 cm/s, which was within the normal range of vascular elasticity for her age.

**Figure 2:**
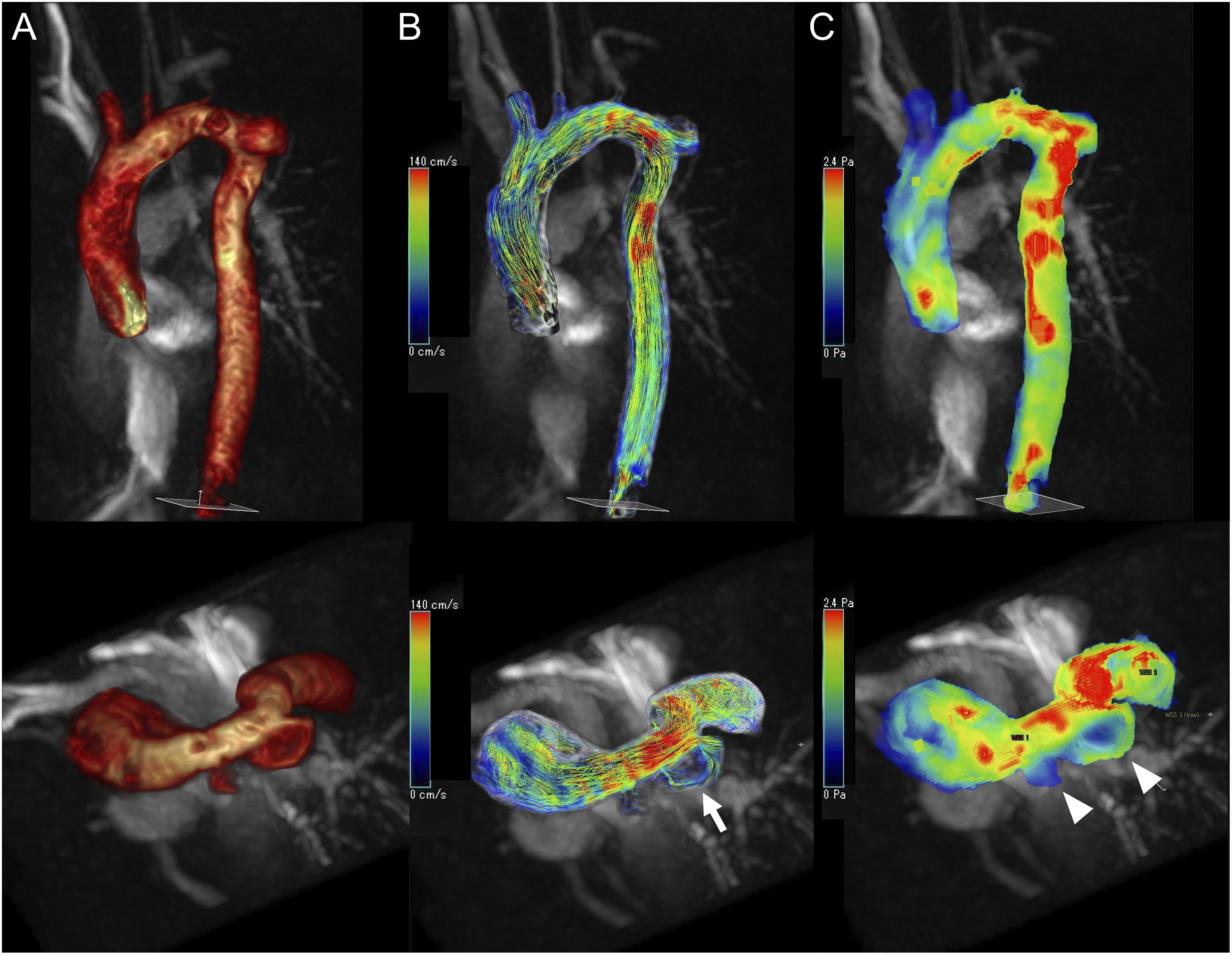
Fig. 2 (**A**) The aortic three-dimensional segmentation image. (**B**) Streamline analysis visualization showing a high-flow pattern in the aortic arch and a vortex into the aneurysm (white arrow) during systole. (**C**) The wall shear stress (WSS) map showing increased peak WSS in the proximal region of the kinking aorta and low WSS in the aneurysms (white arrowheads). (Upper section, left anterior oblique view; lower section, cranial view).

**Figure 3:**
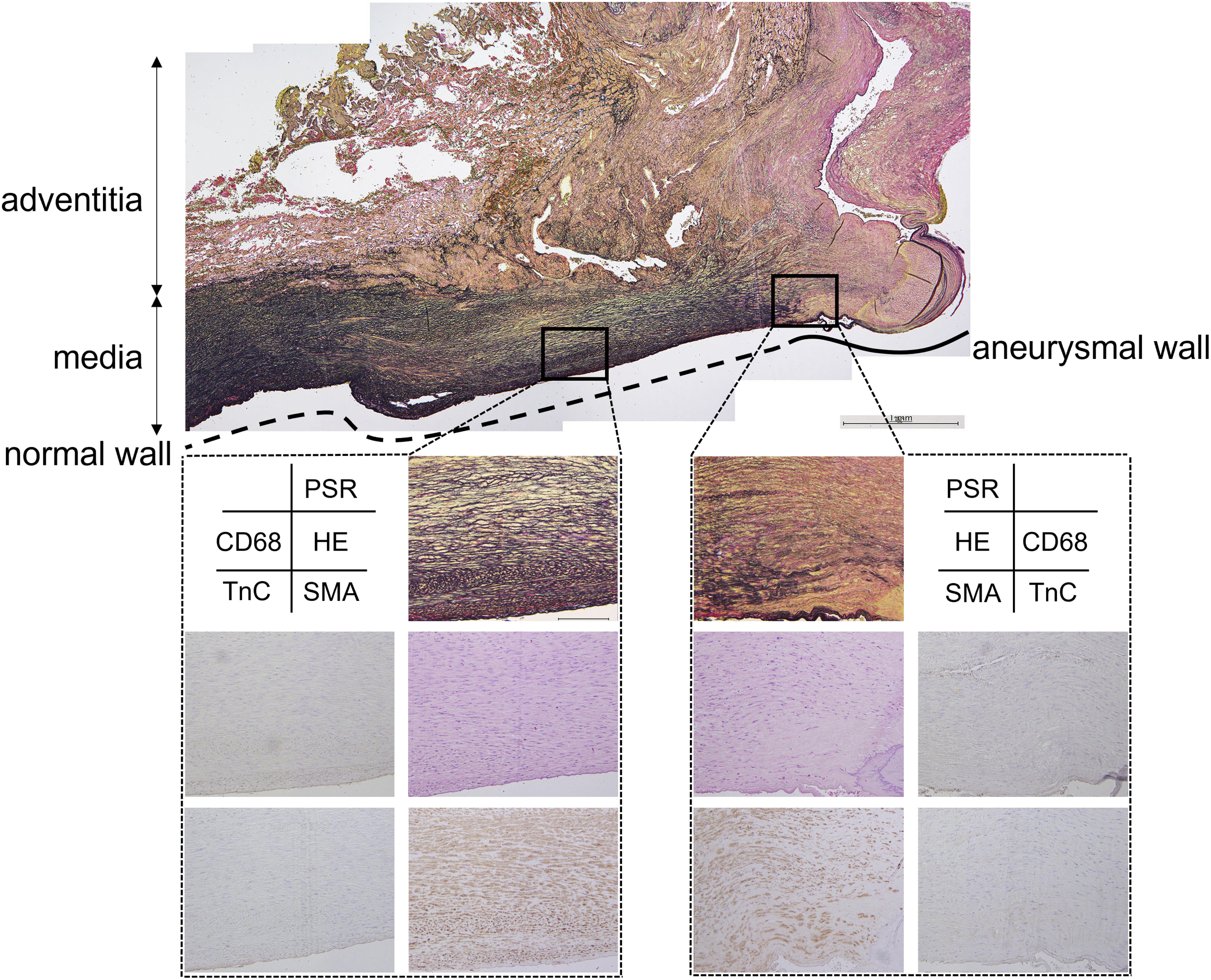
Fig. 3 Pathological findings of a saccular pseudoaneurysm. (Upper) Picrosirius red staining (PSR) of the transition between the normal aorta (dot line) and aneurysmal wall (solid line). Scale bar: 1 mm. (Lower) High-power field images of the normal aorta and aneurysmal wall. The sections were stained with PSR and hematoxylin-eosin (HE) along with immunostaining by CD68, tenascin-C (TnC), and smooth muscle actin (SMA). Scale bar: 20 µm.

The echocardiogram showed a normal ejection fraction and a tricuspid aortic valve without any other congenital cardiac abnormalities. 4D flow MRI was performed to analyze the effect of blood flow on the TAAs and evaluate the hemodynamics. A 3.0 Tesla magnetic resonance scanner (Ingenia 3.0T, Philips Healthcare, Best, Netherlands) was used with respiratory and cardiac gating techniques for the analysis (Supplementary Table). A dedicated analysis software (cvi42, Circle Cardiovascular Imaging Inc., Calgary, Canada) was used to visualize intra-aortic flow. The aortic 3D segmentation was obtained using the magnitude images of the 4D flow MRI data (**Fig. 2A**). Flow patterns in the aorta and aneurysms were visually evaluated from the streamlines. High-velocity flow was observed in the aortic arch near the aneurysms, and the vortex flow completely occupied the entire aneurysm sacs (**Fig. 2B** and **Video 1**). High peak wall shear stress (WSS) was observed in the proximal region of the kinked aorta, whereas lower WSS was observed in aneurysm sacs (**Fig. 2C** and **Video 1**). Therefore, the aneurysms were considered to be at high risk of rupture, and an aortic replacement with a vascular prosthesis was performed for the treatment of TAAs. The patient had an uneventful postoperative course. Histopathological examination of the aneurysmal wall showed that the normal layers of smooth muscle cells and elastic fibers of the media disappeared, while all three layers were replaced with collagen-rich connective tissues (**Fig. 3**). Immunohistochemical staining showed no inflammatory cell infiltration (i.e., CD68-positive macrophages) or expression of tenascin-C, which is an inflammatory/remodeling marker,^4)^ in the aneurysmal and adjacent aortic walls (**Fig. 3**).

## Discussion

In this study, a female patient with PCOA was presented with saccular aneurysms that were assessed by 4D flow MRI and histological analysis. The 4D flow MRI findings revealed the high-velocity flow in the aortic arch near the aneurysms and complete occupation of aneurysm sacs by vortex flow. Peak WSS was high in the proximal region of the kinked aorta, whereas it was lower in the aneurysm sacs. It is noteworthy that the patient had no history of major trauma, arteriosclerotic, and inflammatory lesion in the aneurysm sacs. Therefore, it was suggested that abnormal flow dynamics and resultant abnormal shear stress in the aorta may play a vital role in the development of saccular pseudoaneurysm.

Kimura et al. reported that saccular aneurysms with PCOA may occur in the aortic arch at an early age even in the asymptomatic patients.^5)^ They also reported the partial thinning of aneurysmal wall similar to our case. A previous study demonstrated that the elevated WSS values were associated with a reduced elastin levels and lower smooth muscle cells numbers. Thereby, it can be suggested that WSS may contribute to aortic wall degradation and formation of the ascending TAA.^6)^ In fact, it was recently reported that high WSS levels might be involved in the aorta dilatation.^3)^ Similarly, the observed elevated flow velocity in the proximal area of the kinked aorta in this study was consistent with the previous reports.^7,8)^ It has been reported that WSS has a strong correlation with the velocity of blood flow in the thoracic aorta.^9)^ The elevated WSS values might partially lead to the vulnerability of the aorta that allows the unconscious minor external force to cause an asymptomatic rupture and lead to the formation of pseudoaneurym.

Boyd et al. reported that the rupture risk of abdominal aortic aneurysms is in direct correlation with the WSS level in a region.^10)^ Furthermore, Natsume et al. reported that a vortex flow pattern in the TAA was observed by 4D flow MRI. In their research, low peak WSS was associated with the sac depth/neck width ratio, and peak WSS was invariably low when this ratio exceeded 0.8 in saccular aneurysmal cases.^2)^ These findings were also consistent with the present case. Hence, considering the threat of rupture, the aneurysms were surgically treated. Notably, few reports have worked toward the estimation of TAA rupture risk. Additionally, the relation between the hemodynamic factors and the time course of aneurysmal changes in PCOA has not been studied yet. To predict the progression or rupture risk of TAAs in PCOA, further studies and more data are necessary. Serial noninvasive 4D flow MRI evaluations might help in predicting the occurrence of aneurysms in patients with earlier stages of PCOA.

## Conclusion

It is important to study the development of pseudoaneurysm and the risk of rupture even in young patients with PCOA. Assessment of the kinked aorta in PCOA by 4D flow MRI might be useful in predicting the mechanism of aneurysmal formation.

## Supplementary Information

Supplementary table and movie are available at the online article sites on J-STAGE and PMC.
