# Supplementary material for: Assessment of Pseudocoarctation of the Aorta with Saccular Aneurysms by Four-Dimensional Flow Magnetic Resonance Imaging and Histological Analysis
Source: Ann Vasc Dis. 2022 Dec 25;15(4):348–51. doi: 10.3400/avd.cr.22-00077 (PMC9816040; doi:10.3400/avd.cr.22-00077)
Supplement: Supplementary Data [file avd-15-4-cr.22-00077-s001.pdf]

SUPPLEMENTAL MATERIAL

Supplementary Table. 4D-flow MRI acquisition parameters

4D-flow MRI (3.0 Tesla) acquisition parameters	
Spatial resolution (mm^3)	1.0 × 1.0 × 1.3
Temporal resolution (ms)	54
Field of view (mm^2)	350 × 350
Number of slices	90
TR (ms)	6.9
TE (ms)	4.0
Flip angle (°)	8
Range of velocity encoding (cm/s)	±200
View per cardiac phase	14
Parallel reduction factor	3
gating	respiratory and cardiac

Video 1. Summarized CT and 4D-flow MRI examinations of the saccular aneurysms and kinking of the descending aorta.
